# Quantification of Intra-Muscular Adipose Infiltration in Calf/Thigh MRI Using Fully and Weakly Supervised Semantic Segmentation

**DOI:** 10.3390/bioengineering9070315

**Published:** 2022-07-14

**Authors:** Rula Amer, Jannette Nassar, Amira Trabelsi, David Bendahan, Hayit Greenspan, Noam Ben-Eliezer

**Affiliations:** 1 Department of Biomedical Engineering, Tel Aviv University, Tel Aviv 6139001, Israel; rolaamer@mail.tau.ac.il (R.A.); jannette2210@gmail.com (J.N.); 2 Center for Magnetic Resonance in Biology and Medicine, Aix Marseille University, 13007 Marseille, France; amira.trabelsi.tn@gmail.com (A.T.); david.bendahan@univ-amu.fr (D.B.); 3 Sagol School of Neuroscience, Tel Aviv University, Tel Aviv 6139001, Israel; 4 Center for Advanced Imaging Innovation and Research (CAI2R), New York University School of Medicine, New York, NY 10016, USA

**Keywords:** muscle segmentation, MRI, quantitative MRI, qMRI, T_2_ mapping, deep learning

## Abstract

**Purpose:** Infiltration of fat into lower limb muscles is one of the key markers for the severity of muscle pathologies. The level of fat infiltration varies in its severity across and within patients, and it is traditionally estimated using visual radiologic inspection. Precise quantification of the severity and spatial distribution of this pathological process requires accurate segmentation of lower limb anatomy into muscle and fat. **Methods:** Quantitative magnetic resonance imaging (**qMRI**) of the calf and thigh muscles is one of the most effective techniques for estimating pathological accumulation of intra-muscular adipose tissue (**IMAT**) in muscular dystrophies. In this work, we present a new deep learning (**DL**) network tool for automated and robust segmentation of lower limb anatomy that is based on the quantification of MRI’s transverse (T_2_) relaxation time. The network was used to segment calf and thigh anatomies into viable muscle areas and IMAT using a weakly supervised learning process. A new disease biomarker was calculated, reflecting the level of abnormal fat infiltration and disease state. A biomarker was then applied on two patient populations suffering from dysferlinopathy and Charcot–Marie–Tooth (**CMT**) diseases. **Results:** Comparison of manual vs. automated segmentation of muscle anatomy, viable muscle areas, and intermuscular adipose tissue (IMAT) produced high Dice similarity coefficients (DSCs) of 96.4%, 91.7%, and 93.3%, respectively. Linear regression between the biomarker value calculated based on the ground truth segmentation and based on automatic segmentation produced high correlation coefficients of 97.7% and 95.9% for the dysferlinopathy and CMT patients, respectively. **Conclusions:** Using a combination of qMRI and DL-based segmentation, we present a new quantitative biomarker of disease severity. This biomarker is automatically calculated and, most importantly, provides a spatially global indication for the state of the disease across the entire thigh or calf.

## 1. Introduction

**Figure 1:**
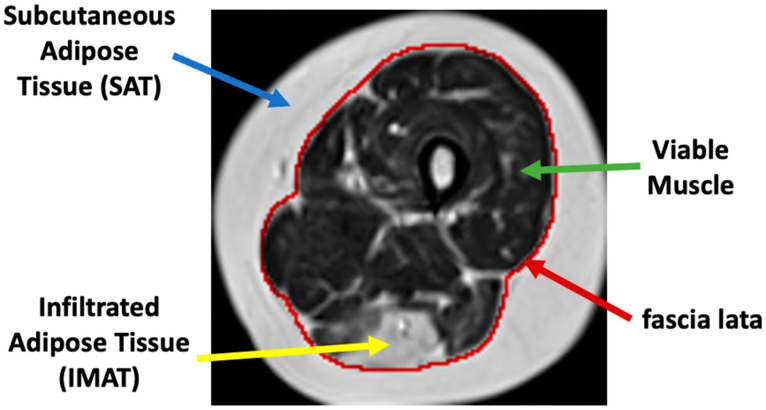
Axial MR image of the thigh. Red: fascia lata boundary; blue, green, and yellow arrows mark the subcutaneous fat, a region of viable muscle, and IMAT pixels.

Muscle dystrophies (**MDs**) are an inherited class of disorders characterized by progressive muscle weakness that affects the upper and lower limbs, the axial muscles, and the facial muscles at variable levels of severity. Fat infiltration into muscles of the lower limbs is one of the hallmarks of these diseases’ progression and can be easily seen in MRI images. MDs lead to a loss of muscle mass and a weakening of muscle strength [1]. The infiltrated fat is usually referred to as intermuscular adipose tissue (**IMAT**) and is separated from the subcutaneous adipose tissue (**SAT**), which surrounds the muscle. The two fat tissues are separated by a boundary layer called “fascia lata”, used in many studies to achieve reliable segmentation of the lower limb anatomy (see Figure 1).

Several MRI modalities have been evaluated for the quantification and assessment of fat infiltration in MD patients [2,3]. These show that MRI-based quantification has a strong correlation with a disease’s progression and can therefore act as an accurate biomarker of disease state and severity as well as improve the prognosis of patients [4,5]. Providing physicians with such a biomarker, however, requires accurate segmentation of muscle tissue, the subcutaneous fat, and the IMAT. This will enable physicians (as well as automated tools) to provide focused assessment of the viability of remaining muscle tissue across the entire calf and thigh anatomies.

IMAT tissue may have a similar image intensity as subcutaneous fat and, hence, sophisticated methods are required to effectively differentiate between these two tissues. The “fascia lata” can also be obscured and hard to find. Other challenges of segmentation techniques include MRI artifacts caused by nonideal hardware, natural tissue heterogeneities, spatial bias of images’ intensity, and motion artifacts, to name but a few [6].

In this work, we introduce a new muscle-specific index (or biomarker), reflecting the pathological stage of the muscle and, specifically, the ratio between the IMAT area and the entire muscle region. The study presents two main contributions: the first is a new neural network that can automatically identify and discard the SAT, the calf and thigh bones, and the bone marrow pixels, leaving only the muscle region. We then show that the muscle region segmented by the network can be further classified into viable muscle pixels and IMAT pixels based on a quantitative measurement of MRI’s transverse (T_2_) relaxation times. Application of the new pipeline and quantitative biomarker is presented for two muscle dystrophies: dysferlinopathy and Charcot–Marie–Tooth (**CMT**), exhibiting similar patterns of fat infiltration.

### 1.1. Related Work

In this section, several works related to the aims of this study are reviewed. These include traditional tools for segmentation of calf and thigh anatomies, weakly supervised learning, and deep convolutional auto-encoders with clustering.

#### 1.1.1. Segmentation and Quantification of Epicardial Adipose Tissues (EAT)

Adipose tissues play an important role in human function. The literature has shown correlations between epicardial tissue and obesity as well as other diseases such as coronary atherothrombotic diseases. Several works utilizing semi-automated [7] and deep-learning-based automated schemes [8] can be found that segment the EAT from coronary computed tomography angiography (CCTA). In addition to segmentation of EAT regions, quantification of information is shown, with several important measures extracted, including fat densities distribution, enabling in-depth study towards a possible correlation between fat amounts, fat distribution, and heart diseases.

#### 1.1.2. Calf and Thigh Segmentation

Several works have been published for segmentation of thigh and calf anatomies. Most studies utilized conventional unsupervised methods including k-means, fuzzy c-means, active contours, and Gaussian mixture model-expectation maximization (GMM-EM) to segment subcutaneous fat, muscle, intermuscular fat, and bones [9,10,11]. The main problem facing these approaches is that segmentation using active contour-based methods yields unreliable results when the “fascia lata” is obscured, which usually is the case in moderate to severe disease diagnoses. Other works introduced a muscle region segmentation method by detecting the facia lata contour [12,13,14]. Advancements have also been shown using deep learning convolutional neural networks (**CNNs**), used for automatic segmentation of IMAT in thigh and calf MR images. Yao et al. [15] integrated deep-learning logic with traditional models, proposing a holistic CNN and dual active contour model for detection of fascia lata and classification into muscle and IMAT. In the current work, we addressed the task of finding contours in pathological cases by generating muscles’ masks using a convolutional network, thus assuring that the anatomical and textual information were learned for both viable muscle and for tissue that underwent fat infiltration.

#### 1.1.3. Weakly Supervised Learning

Many works have been published on limited data annotations. Weakly supervised learning was used for object detection and classification in optical remote sensing and satellite images through features learning and image-level labels [16,17,18]. Weakly supervised learning has also attracted researchers in the medical image domain. Such methods have been used for multiple tasks in medical images such as image classification, semantic segmentation, and patch-level clustering [19,20,21]. Weak learning is performed using image scribbles, image-level labels, partial training data annotations, and recursive training. In this work, weak supervision is utilized in order to solve the uncertainty problem in labeling of muscle tissue in muscle dystrophy patients for the task of patches clustering.

#### 1.1.4. Clustering with Convolutional Auto-Encoder

Convolutional neural networks have been widely used for classifying medical images into normal and diseased conditions [22,23] using annotated images. The lack or sparse annotations of data, however, raises the need for unsupervised methods such as convolutional auto-encoders. Masci et al. [24] presented the convolutional auto-encoder for the first time. The convolutional auto-encoder is an unsupervised method for hierarchical feature extraction. The extracted features can be exploited for clustering by simply applying k-means or other clustering methods on the extracted features [25]. Recent results demonstrated that combining the separated stages in a unified framework and training them jointly in an end-to-end way can achieve better performance [26,27]. In this work, a convolutional auto-encoder was trained to extract features from patches cropped from fat and viable muscle tissues, followed by applying k-means in the embedded space to find two clusters. Our results were also compared to a technique that trains a convolutional auto-encoder and clustering layer in an end-to-end way.

## 2. Methods

**Figure 2:**
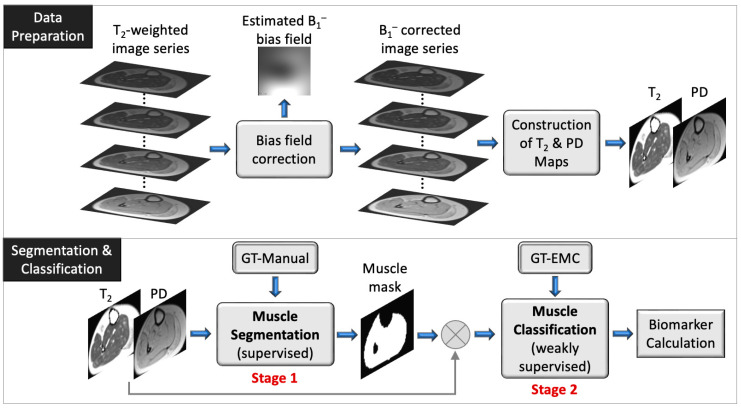
Schematic overview of data preparation (top) and segmentation and classification pipeline (bottom). The muscle segmentation (stage 1) and muscle classification (stage 2) are also described in the Supplementary Materials. The biomarker is defined by Equation (4). GT: ground truth; PD: proton density.

The overall processing pipeline presented in this work is depicted in Figure 2. We start with a description of the two main processing stages of the proposed solution.

In the first stage we detect and discard the SAT, the calf and thigh bones, and the bone marrow pixels, leaving only the muscle region. The fascia lata serves as a visual separator, creating a reliable ground truth (**GT**) that enables supervised learning methods to accomplish this task precisely. Encouraged by its high efficiency in semantic segmentation of small datasets, a U-net architecture was thus employed for segmenting the muscle region at this stage. A good performance was achieved for various levels of pathology and, especially, for severe cases of fat infiltration, which are the most challenging to segment.

The second stage of analysis discriminated between viable muscle pixels and IMAT pixels residing within this muscle region. Pixels were classified into these two categories based on their quantitative T_2_ relaxation times and sub-pixel fat fraction. The uncertainty in the ground truth labeling for this task motivated us to use a weakly supervised approach. Pixels with fat infiltration can be randomly dispersed over the entire muscle tissue with variable levels of infiltration. Consequently, the border between viable muscle and IMAT pixels becomes blurry and less defined, making manual segmentation difficult and uncertain. Hence, a weakly supervised method constitutes a more natural choice for this stage. Inspired by [28], a patch-based deep convolutional auto-encoder with a triplet loss constraint was implemented to learn an interpretable latent feature representation and apply k-means in the embedded space in order to classify image pixels into two clusters. This integration of patches helps overcome the problem of small data, while exploiting the contextual information among pixels and maintaining the relationship to adjacent pixels. The presented results demonstrate the key role of clustering in our task and the effectiveness of the overall system in predicting the fat infiltration levels.

Figure 2 provides a schematic illustration of the presented segmentation pipeline. Data are first preprocessed: the receiver coil bias field (B_1_^−^) is corrected for a series of input T_2_ weighted images, followed by generation of quantitative T_2_ and proton density (**PD**) maps using the EMC algorithm [29]. Next (bottom panel), the muscle region is segmented using a supervised method (stage 1) and classification of muscle pixels is made into viable muscle and IMAT using a weakly supervised approach (stage 2). Finally, the infiltrated fat index is calculated, serving as the output biomarker.

### 2.1. MRI Scans

The first dataset included MRI scans of the lower limbs (thigh and calf) from 17 dysferlinopathy patients (36 ± 4 years old). This dataset was used for training the networks and evaluation of stage 1 and stage 2 of the segmentation procedure. Each scan included 5 axial slices, each of which included a time series of 17 T_2_-weighted images, sampled at increasing echo times (**TEs**). Two additional datasets of dysferlinopathy and Charcot–Marie–Tooth disease (**CMT**) patients were used for testing. These datasets included nine dysferlinopathy patients and 15 CMT patients, each containing 10 slices and 17 echo times. MRI scans were performed on a whole-body Siemens Prisma 3T scanner after signing an informed consent and under the approval of the local Helsinki and IRB committees. Quantitative T_2_ maps were generated from a multi-echo spin-echo (**MESE**) protocol using the following parameters: TR/TE = 1479/8.7 ms, N_echoes_ = 17, in-plane resolution = 1.5 × 1.5 mm^2^, matrix size of 128 × 128, N_slices_ = 5, slice thickness = 10 mm, and acquisition time = 5 min 7 s.

### 2.2. Data Preparation

In this section, we describe the preprocessing steps performed for bias field removal, generation of T_2_ and PD maps, and the generation of GT labeling.

#### 2.2.1. Bias Field Removal

**Figure 3:**
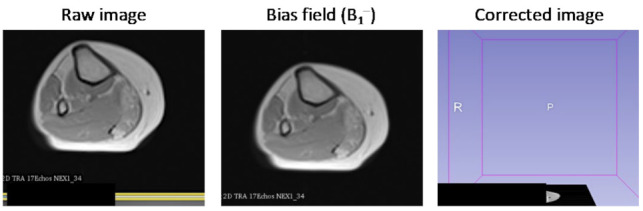
Correcting receiver gain bias in an axial MRI of the calf. Left: Image affected by inhomogeneous B_1_^–^ intensity bias; Middle: bias field, estimated using the N4ITK method; Right: image after intensity correction.

MRI receiver coils have an inherently inhomogeneous spatial sensitivity profiles, causing a bias field (B_1_^−^) in the images. This effect was corrected by using the N4ITK method [30]. The input to this step is a series of T_2_ weighted images, sampled at increasing echo times and for different slice locations, while the correction was applied separately for each slice. The method was implemented in 3D Slicer (http://www.slicer.org accessed on 1 February 2022) [31]. Figure 3 shows an input image (left) that was contaminated with an inhomogeneous bias field (middle) causing a nonphysiological variation in the signal intensity to appear at the bottom of the image. The right panel demonstrates how this bias field can be effectively removed, resulting in a more homogeneous depiction of the imaged anatomy.

#### 2.2.2. Construction of T_2_ and PD Maps

Generation of T_2_ and PD maps was conducted using the echo modulation curve (**EMC**) algorithm described in [29,32]. Reliable quantification of single T_2_ values is a challenging task due to the contamination of fast multi-echo spin echo (**MESE**) protocols by stimulated echoes [33]. The EMC algorithm can overcome this limitation and deliver accurate and reproducible T_2_ maps. Briefly described, this algorithm consists of two steps. First, a precalculated dictionary of theoretical EMC’s is simulated using the time-dependent Bloch equation. Each simulation generates a single EMC, designating the intensity of the MESE decay curve for a specific pair of T_2_ and transmit field (B_1_^+^) values. A full dictionary is then constructed by repeating the simulations for a range of T_2_ and B_1_^+^ values. Once a dictionary is prepared, quantitative T_2_ values of the tissue are extracted on a pixel-by-pixel basis by matching the experimental decay curve at each imaged pixel to the precalculated dictionary of simulated EMCs. Matching is performed by searching for the dictionary entry that yields minimal *l*_2_-norm of the difference between experimental and simulated curves. Following this procedure, a unique T_2_ value is assigned to each pixel, yielding the desired parametric map. Finally, a proton density (**PD**) map is calculated by back-projecting the intensity of each pixel in the image from the first echo-time (*t* = TE) to the time point *t* = 0 using the fitted T_2_ map, seeing as a pure exponential decay takes place between the excitation event and the first acquired echo [34].

#### 2.2.3. Preparation of Ground Truth (GT) Data

GT labeling of muscle regions that were utilized for stage 1 training were delineated by a musculoskeletal radiologist with 10 years of experience. GT labeling of IMAT and viable muscle for stage 2 were performed on a pixel-by-pixel basis. In peripheral muscle disorders, the infiltration of fat into the diseased muscle region causes a mixture of two T_2_ components to appear in each imaged pixel. An extension of the EMC algorithm was recently introduced [35], providing a measure of “disease severity” in neuromuscular dystrophies, based on a two T_2_ component fit of the MRI signal. This offers simultaneous estimation of fat and of water fractions at a sub-pixel level. A threshold was thus set, where pixels with fat fraction >50% were labeled as “fat” (i.e., diseased muscle), while the remaining pixels were labeled as viable muscle. The fraction between IMAT and the entire muscle region was then used as a ground truth measure of disease severity.

### 2.3. Stage 1: Muscle Region Semantic Segmentation

In the first analysis stage, a neural network was trained to segment the region inside the “fascia lata”. The subcutaneous adipose tissue (SAT), bone, and bone marrow were automatically masked out in this method.

#### 2.3.1. Network Architecture

A common fully convolutional network (**FCN**)-based deep learning U-net architecture was employed for the segmentation of the muscle region [36]. This U-net has been shown to perform well on medical images with very few learning samples and strong use of data augmentation. The encoder part of the network is a contracting path, while the decoder part is a symmetrical expanding path that decompresses the features back to their original size. The concatenating path consists of five levels with different resolution feature maps. Each level consists of two layers of 3 × 3 nonpadded convolutions followed by a rectified linear unit (**ReLU**). Following the two convolution layers, a 2 × 2 max pooling operation is applied with stride 2 for down-sampling. After each down-sampling step, the number of feature channels is doubled in the next two convolution layers. The expansion path consists of five levels in which the number of feature channels is repeatedly halved. In each level, a transposed convolution (i.e., deconvolution) is used, with a 2 × 2 kernel size and stride of 2. The transposed convolution optimally learns the up-sampling, which helps restore the image more precisely than using interpolation for up-sampling. Next, the maps are concatenated with the corresponding feature maps from the contracting path and then two 3 × 3 convolutions are applied, each followed by a ReLU. At the final layer, a 1 × 1 convolution is used to map the feature vector to the desired number of classes.

#### 2.3.2. Network Inputs

Seven types of inputs into the FCN were considered in this work. In two cases, inputs were comprised of T_2_ and PD maps (2 channels)—once without preprocessing and once after correcting the map’s inhomogeneous intensity and cropping the images around the anatomy boundary. Four additional inputs included the use of unprocessed or processed T_2_ maps only, or the use of unprocessed or processed PD maps only. A seventh FCN was trained on a series of 17 raw T_2_-weighted images that were acquired at increasing echo times and then used to generate the T_2_ and PD maps. The purpose of these experiments was to explore the FCNs’ ability to produce sufficient results in the existence of MRI artifacts and in the absence of inhomogeneity correction methods, and, secondly, to test the hypothesis that training a network using quantitative maps is equivalent to training it using raw data, on which these maps are based.

#### 2.3.3. Training Procedure

The training set included data from 14 patients along with their corresponding binary segmentation maps. The thigh/calf anatomies were first separated from the images’ background using a canny edge detector, enabling accurate delineation of the tissue’s outer edge. Then, images were cropped around the region of interest and resized to 128 × 128. Suspected outlier values in individual pixels were corrected in the T_2_ maps by taking the 98% percentile of the image’s dynamic range and clipping pixels above this value. Finally, each image was normalized to have a zero mean and unit variance before training the network. Adam optimizer [37] was used in the model training, with the parameters: *lr* = 0.001, *β*_1_ = 0.9, *β*_2_ = 0.999, and *ε* = 10^−8^. The optimized loss function was a soft Dice coefficient loss. A size of 8 was selected for the batch size. Training was conducted for 100 epochs. Augmentation of the original training data was conducted, increasing the number of images by a factor of 10 and improving the model’s robustness in the presence of data variance. Several augmentations strategies were used including a shift (0.2 of image height and width), zoom (between 0.9 and 1.3 of image size), rotation (0°–30°), and flip (vertical/horizontal). The proposed method was implemented in Python and the keras library. The training process was performed on a desktop PC with an NVIDIA GeForce GTX 1080 Ti GPU.

### 2.4. Stage 2: Classification into Viable Muscle and IMAT

In this stage, we further classified the muscle pixels into two types: viable muscle pixels and IMAT pixels. A patch-based deep convolutional auto-encoder (**DCAE**) was employed to learn semantic feature representation incorporating deep metric learning. The goal we set forth was to learn an embedding for the patch-level feature representation to enable tissue clustering using an unsupervised scheme. The constraint we applied was that different tissue patches would be represented as nonsimilar, while same tissue patches will be similar in the selected space.

**Figure 4:**
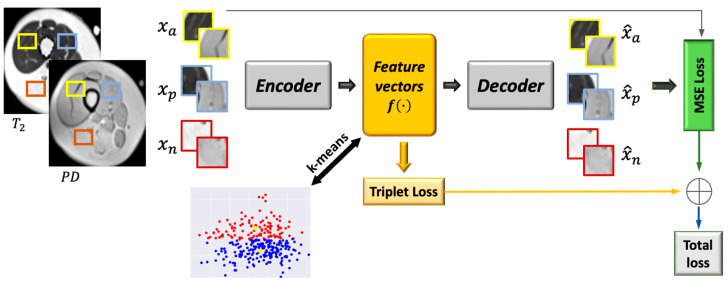
Integration of deep convolutional auto-encoder and triplet loss with k-means. See text for a more elaborate description of the network architecture.

A general description of the proposed method is illustrated in Figure 4. Three pairs of patches were used as input to the network: (i) two patches cropped from the proton density and T_2_ maps; (ii) two inputs sampled from one type of tissue (e.g., IMAT); (iii) one sample from the second tissue (e.g., viable muscle). The encoder transforms these patches into feature representation, while the decoder reconstructs the original patches from the feature vectors. The objective function consisted of two terms: regularization of the mean squared error (**MSE**) loss in order to penalize the autoencoder on the reconstructed patches and avoid data collapse; triplet loss that is computed on the feature vectors and imposes similarity between patches of the same tissue and dissimilarity between patches of different tissues. Once the network was trained, the features space was constructed using the training data, followed by applying k-means to obtain two clusters. A label was assigned to each pixel, followed by labeling the entire cluster based on the labeling of the majority of pixels in that cluster. In the test phase, the input patch was classified to the closest cluster.

#### 2.4.1. Patch Cropping

The network input was composed of two maps, T_2_ and PD, which were multiplied by the muscle region mask generated from the first stage to produce muscle-only regions. The classification was performed on patches cropped around each pixel that had the same label as the central pixel. The patches were cropped from both T_2_ and PD maps and concatenated into 2 channels. The size of each patch was 16 × 16. A challenging issue in this stage was the boundary of the muscle region: patches cropped from the boundary can have different features than patches cropped from the center of the muscle region and may be clustered into a distinct cluster. To overcome this issue, morphological erosion with a 16 × 16 kernel size was applied to the muscle region mask and the coordinates of the positive pixels in the eroded mask were calculated; then, the patches around these coordinates were cropped from the T_2_ and PD maps (that were multiplied by the uneroded mask) to avoid boundary-region patches altogether. Following classification, the mask was dilated with the same kernel to include the pixels that were removed.

#### 2.4.2. Deep Convolutional Auto-Encoder and Triplet Loss (DCAETL)

**Figure 5:**
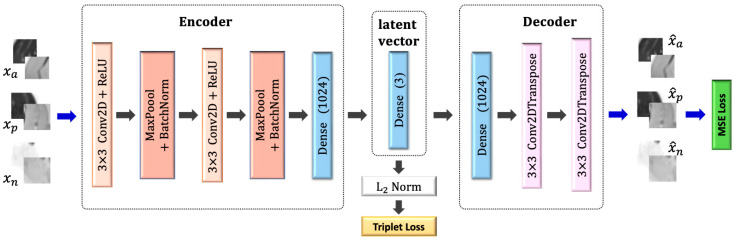
Schematics of the deep convolutional auto-encoder architecture and the loss terms used for the automatic classification of muscle tissues.

Figure 5 shows schematics of the deep convolutional auto-encoder architecture and the loss terms used for the automatic classification of muscle tissues. DCAE, consisting of an encoder and a decoder, was trained on patches of 16 × 16 × 2 in size extracted around each pixel from the T_2_ and PD images of the muscle region. The encoder consisted of two blocks having 32 and 64 feature maps. Each block is built of 3 × 3 convolutions followed by ReLU, 2 × 2 max pooling, and batch-normalization. The output of the two fully convolution blocks was then flattened into 1024 units and followed by a dense layer that encoded the features in the embedded space. A normalization step followed (L2) to constrain the embedding to a hypersphere. The decoder structure was composed of a dense layer of 1024 reshaped to size 4 × 4 × 64, followed by 3 × 3 transposed convolution layer with 32 filters and stride 2, and another 3 × 3 transposed convolution layer that reconstructs the input patch. The DCAE was trained with Adam optimizer with default settings and a batch size of 256 for 100 epochs.

Two components comprised the loss function: a reconstruction loss and a triplet loss. The reconstruction loss is the mean squared error (**MSE**) expressed in Equation (1). To train, the input patch triplet (i.e., $x_{a}^{i}$, $\;x_{p}^{i}$, and $\;x_{n}^{i}$) was randomly selected from the training set, and the anchor was randomly selected from all the cropped patches in the training set. The label of the anchor was then checked, and the positive and negative patches were determined according to the label of the anchor. If the label of the anchor was 1 (i.e., viable muscle), then the positive patch was randomly selected from the patches with label 1 (viable muscle patches); otherwise, the negative patch from the patches with label 0 (IMAT patches) was selected. If the anchor had a label of 0 (IMAT), then the positive and negative patches were selected accordingly (i.e., positive—IMAT, negative—viable muscle). The distance of the anchor patch ($x_{a}^{i}$) from the positive patch ($x_{p}^{i}$) that roughly matched the anchor patch was smaller than the distance from the negative patch ($x_{n}^{i}$). The auto-encoder was trained simultaneously on the three patches, transforming them into latent vectors, $f\left(x_{a}^{i}\right)$, $f(x_{p}^{i}$), and $f\left(x_{n}^{i}\right)$, and reconstructing each one into the original image (i.e., ${\hat{x}}_{a}^{i}$, $\;{\hat{x}}_{p}^{i}$, and $\;{\hat{x}}_{n}^{i}$). The latent vectors were used for the calculation of the triplet loss (Ltriplet). The triplet loss over a batch N can be expressed as follows:

(1) $$L_{MSE}\left(x,\hat{x}\right)=\frac{1}{N}\overset{N}{\underset{i=1}{\sum }}{\left(x^{i}-{\hat{x}}^{i\;}\right)}^{2}$$

(2) $$L_{triplet}=\sum_{i=1}^{N}\max\left\{0,\|f\left(x_{a}^{i}\right)-f{\left(x_{p}^{i}\right)\|}_{2}^{2}-f\left(x_{a}^{i}\right)-f{\left(x_{n}^{i}\right)\|}_{2}^{2}+\alpha \right\}$$

where *α* is a margin enforced between positive and negative pairs and set to 1 in our experiments. The combined loss is defined in Equation (3):

(3) $$L_{total}=\beta L_{triplet}+\lambda (L_{MSE\;}\left(x_{a},{\hat{x}}_{a}\right)+L_{MSE}\left(x_{p},{\hat{x}}_{p}\right)+L_{MSE\;}\left(x_{n},{\hat{x}}_{n}\right)$$

Here, *β* and *λ* denote loss weights, which were experimentally set to one-half and one-sixth, respectively.

### 2.5. Quantification of IMAT Biomarker

To quantify the fraction of infiltrated fat, the muscle region was segmented from thigh/calf MR images in stage 1; then, muscle region pixels were classified to viable tissue and IMAT in stage 2. The IMAT fraction was computed based on the muscle region area, which was the pixels’ sum (*Area_whole−muscle_*) and the sum of IMAT pixels (*Area_IMAT_*), using the following equation:

(4) $$IMAT\;\;fraction=\frac{Area_{IMAT}}{Area_{whole-muscle}}$$

This biomarker was calculated for each image and indicated the relative fraction of nonviable tissue (in our case, where pixels contained more than 50% fat).

### 2.6. Performance Evaluation

Performance evaluation was performed by comparing the different stages of the proposed system to corresponding schemes in the literature. The results from stage 1 were compared with the *ASeg* method described in [38]. The *ASeg* method can briefly be described as follows: k-means clustering is applied to the intensity values in order to segment the image to background, muscle tissue, and adipose tissue. In a second step, *ASeg* performs morphological closing to eliminate noise and merge muscle and adipose tissues. Next, a polygonal active contour is constructed in order to define the boundaries between the muscle, bone, and SAT.

The DCAETL method in Section 2.4.2 was compared to the following methods: intensity based k-means, where the simple k-means algorithm was applied to the intensity values of each pixel in the T_2_ and PD maps; deep convolutional auto-encoder followed by k-means; deep convolutional auto-encoder with deep clustering. A detailed description of each of these methods is described below.

#### 2.6.1. Deep Convolutional Auto-Encoder Followed by k-Means (DCAE + k-Means)

The deep convolutional auto-encoder had the same architecture as described in Figure 4. The encoder, decoder, and latent vector shared the same number of layers and filters. The difference was in the input and objective functions. The input to the DCAE was one patch cropped randomly from the T2 and PD maps. The DCAE was trained on the patches with the MSE loss only. The embedded feature space was constructed from the feature vectors, and k-means was applied to the embedded space to obtain two clusters (i.e., infiltrated fat and viable muscle). The DCAE was trained with the Adam optimizer using default settings and a batch size of 256 for 100 epochs.

#### 2.6.2. Deep Convolutional Auto-Encoder with Deep Clustering (DCAE_DC)

Here, we followed a widely used training scenario, i.e., the DCAE was first pretrained and then fine-tuned with clustering-oriented loss. The method was motivated from [26], with the training paradigm conducted in two steps:

(1) Initialization of the DCAE

The parameters of the network were initialized by training the DCAE depicted in Figure 4 on patches cropped from T_2_ and PD maps. Prior to training the network end-to-end, k-means was performed on the outputs of the bottleneck layer of the pretrained DCAE to obtain initial values of the clusters’ centers. The DCAE was pretrained with the Adam optimizer using default settings and a batch size of 256 for 1 epoch.

(2) Deep convolutional auto-encoder embedded clustering

**Figure 6:**
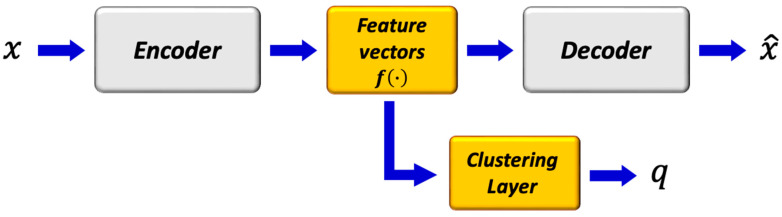
Structure of deep convolutional embedded clustering. The architecture consists of convolutional auto-encoders and a clustering layer connected to the embedded layer of the auto-encoders.

The structure of the network is shown in Figure 6. The encoder, decoder, and feature vector had the same number of layers and parameters as in Figure 5, while the clustering layer maps each embedded vector, z, of input patch, *x,* into a soft label, *q*. Then, the clustering loss, *L_c_*, was defined as the Kullback–Leibler divergence (KL divergence) between the distribution of soft labels, *q,* and the predefined target distribution, *p*. The objective of the network is:

(5) $$L=L_{r\;}\left(x,\;\hat{x}\right)+\gamma L_{c\;}\left(q\right)$$

where *L_r_* and *L_c_* are the reconstruction loss and clustering loss, respectively, and γ > 0 is a coefficient that controls the degree of the embedded space distortion. The clustering layer maintains cluster centers ${\left\{{µ}_{j}\right\}}_{1}^{K}$ as trainable weights and maps each embedded point *z_i_* into soft label *q_i_* using the Student’s *t*-distribution [39]:

(6) $$q_{ij}=\frac{{\left(1+\|z_{i}-\mu_{j}\|{}^{2}\right)}^{-1}}{\sum_{j}{\left(1+\|z_{i}-\mu_{j}\|{}^{2}\right)}^{-1}}$$

where *q_ij_* is the *j*th entry of *q_i_*, representing the probability of *z_i_* belonging to cluster *j*. The clustering loss is defined as:

(7) $$L_{c}=KL\left(P||Q\right)=\underset{i}{\sum }\underset{j}{\sum }p_{ij}log\frac{p_{ij}}{q_{ij}}$$

where *P* is the target distribution, defined as

(8) $$p_{ij}=\frac{q_{ij}^{2}/\sum_{i}q_{ij}}{\sum_{j}(q_{ij}^{2}/\sum_{i}q_{ij})}$$

The *γ* = 0.1 parameter was set, and the DCAE’s weights, cluster centers, and target distribution, *P*, were fine-tuned. The target distribution, *P*, serves as the GT soft label but also depends on the predicted soft label. Therefore, to avoid instability, *P* should not be updated at each iteration using only a batch of data. The target distribution was updated using all embedded points every T = 200 iterations. The training process was terminated if the change of label assignments between two consecutive updates for target distribution was less than a threshold of δ = 0.0001.

#### 2.6.3. Metrics

The segmentation performance was evaluated using the Dice similarity coefficient (DSC) of the predicted delineation to the GT annotation.

(9) $$\mathrm{DSC}=\frac{2\left|X\cap Y\right|}{\left|X\right|+\left|Y\right|}$$

Three scores were utilized to assess the clustering results: normalized mutual information (**NMI**), accuracy of clustering (**ACC**), and adjusted Rand index (**ARI**). NMI is an information theoretic similarity score that assesses the mutual information of the clusters with the ground-truth classes, with per-class normalization. Given a set of true clusters and the set of clusters found by an algorithm, these sets of clusters must be compared to see how similar or different the sets are. A normalized measure is desirable in many contexts, for example, assigning a value of 0 where the two sets were totally dissimilar, and 1 where they were identical. ARI is a variant index, adjusted for the chance grouping of elements. This index reflects a similarity measure between two clusterings by considering all pairs of samples and by counting pairs that are assigned in the same or different clusters in the predicted and true clusterings. The adjusted Rand index was thus ensured to have a value close to 0.0 for random labeling, independently of the number of clusters and samples, and exactly 1.0 when the clusterings were identical (up to a permutation). ACC was estimated as the ratio between accurate predictions to the total number of predictions conducted by the network:

(10) $$\mathrm{ACC}=\frac{TN+TP}{TN+TP+FN+FP}$$

where *TN* is true negative; *TP* is true positive; *FN* is false negative; *FP* is false positive.

## 3. Results

**Figure 7:**
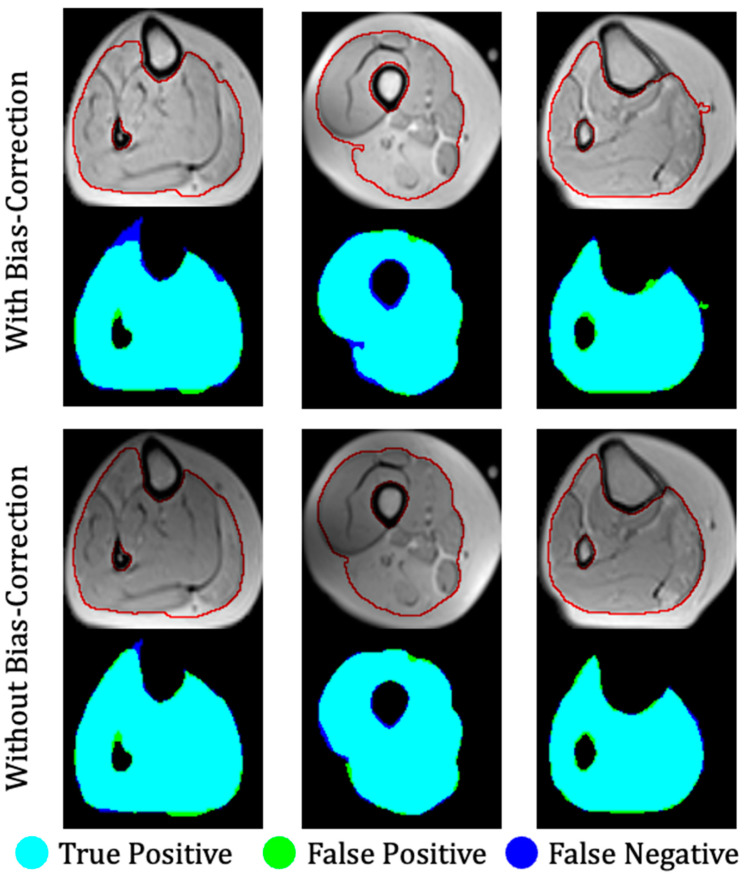
Three examples of segmentation of a calf anatomy. Left to right: mild, moderate, and severe levels of fat infiltration. **Top:** With bias field correction; **bottom:** without bias field correction. Manual segmentation contours are shown in red. Overlap between ground truth and the output of the fully convolutional network is color-coded to indicate regions of true positive, false positive, and false negative segmentation.

The proposed method was evaluated on the first dataset, which included 17 dysferlinopathy patients, with three test patients exhibiting mild, moderate, and severe levels of fat infiltration. Severity was evaluated on a slice-by-slice basis, where the pathology in each slice was considered mild, moderate, or severe according to whether IMAT was in the range 0–33%, 34–66%, or 67–100% of the entire muscle region, respectively. The results for the muscle region segmentation (stage 1) are presented in Table 1 and show a very high performance for the variety of inputs, proving the reliability and robustness of the method. The results are presented for mild, moderate, and severe patients as well as averaged over entire patient set. The results were also introduced using the leave-one-patient-out (**LOPO**) method on the 17 patients and demonstrated the generality and robustness of the method. The results in Table 1 show that the suggested method surpassed the *ASeg* technique at all disease severity levels, while the *ASeg* method performance deteriorated as the disease severity progresses, and our method preserved a high dice coefficient. Figure 7 shows the segmentation results where the muscle region was correctly segmented, even around the fascia lata, with a very small number of pixels that were wrongly classified (i.e., FPs and FNs). Accurate results were achieved, even in cases of strong fat infiltration.

**Figure 8:**
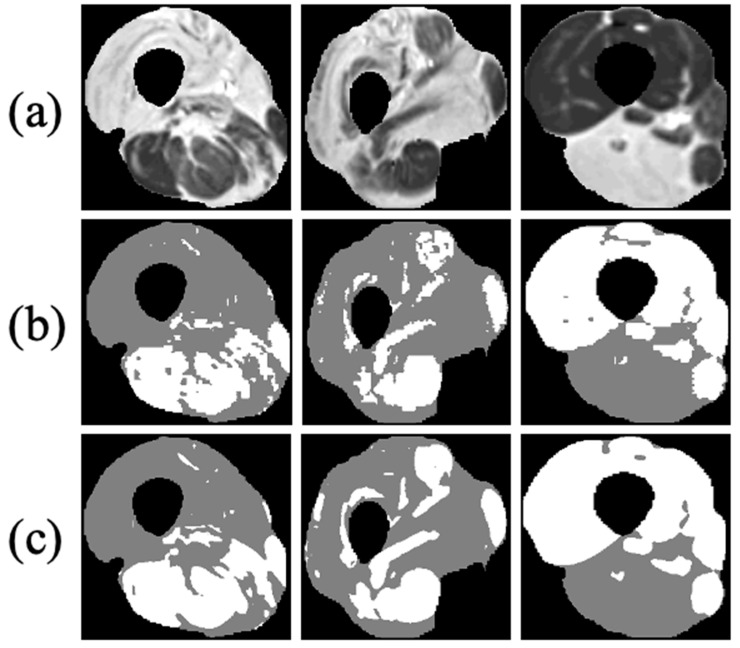
Three examples of FCN-based classification of thigh anatomy: (**a**) axial MR images of three different patients after applying the first stage of the proposed method and segmenting the muscle region; (**b**) ground truth of the intermuscular adipose tissue (**IMAT**) and viable muscle pixels within the muscle region (white: viable muscle: gray: IMAT pixels); (**c**) tissue classification using the suggested FCN processing.

The results of the different clustering methods explored are presented in Table 2 (stage 2 of the postprocessing pipeline). The proposed method—DCAETL with k-means—outperformed the competing methods in viable muscle Dice, IMAT Dice, accuracy, NMI, and ARI. In Figure 8, the visualization results of the clustering of viable muscle and IMAT are shown, the classification was shown only on the muscle region that was segmented in stage 1.

**Figure 9:**
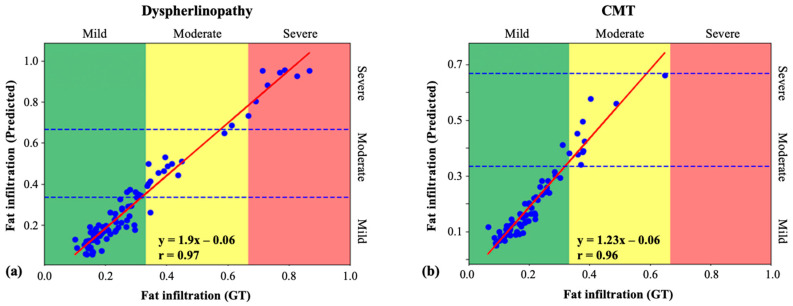
Linear regression of the IMAT fraction biomarker estimated from ground truth and using the suggested FCN technique: (**a**) dysferlinopathy patients’ data; (**b**) Charcot–Marie–Tooth (CMT) patients’ data.

To validate the quantification of infiltrated fat, the method was also tested on larger datasets of 9 dysferlinopathy and 15 CMT patients, with 10 slices per patient. Dysferlinopathy patients exhibited mild, moderate, and severe conditions, while the CMT dataset contained only mild and moderate disease states. The biomarker was computed from the GT masks and the masks segmented by the network according to Equation (4). Table 3 shows the comparison of the average calculated biomarker between GT and predicted values for each disease and severity level. Very close ranges were produced for each biomarker. Figure 9 shows results of linear regression fit on the dysferlinopathy and CMT patients. The high correlation coefficients of 97.7% and 95.9% demonstrate that our calculated biomarker was strongly correlated with the GT, proving that the proposed method is very efficient in quantifying the disease state.

## 4. Discussion

This work presents a deep-learning-based approach for segmentation and classification of muscle and adipose tissues in the calf and thigh anatomy of dysferlinopathy and CMT patients based on quantitative MRI data. This allows for the assessment of disease severity by quantifying the level of fat infiltration in an automated way and across the entire scanned anatomy. The quantification of fat infiltration was performed in two stages: the muscle region was first segmented followed by classification of muscle pixels into viable muscle and fat infiltrated tissues. The well-known U-net architecture was utilized for the muscle segmentation task, and a variation of the convolutional auto-encoder was used to learn semantic features that enabled classification of the muscle tissue.

The quantitative results showed a high segmentation accuracy for dysferlinopathy and CMT patients. A linear regression fit demonstrated high agreement between ground truth and the predicted biomarker with correlation coefficients of 97.7% and 95.9% for the dysferlinopathy and CMT patients, respectively. The separate performance of stages 1 and 2 were also evaluated. Stage 1 achieved a Dice score of 96.4%, and stage 2 achieved scores of 91.7% and 93.3% for classification of the viable muscle area and intermuscular adipose tissue, respectively. A high Dice score of 96.2% was achieved even on patients with high disease severity, demonstrating that the network can detect the fine line (i.e., the “fascia lata”) between subcutaneous fat and infiltrated fat and, consequently, to accurately segment the diseased muscle region. Multiple experiments with different inputs also provide strong support for the U-net’s relevance to the task. An analogous experiment with an original set of raw T_2_-weighted images was meant to justify our selection of quantitative T_2_ and PD maps. Networks that were fed with one or both quantitative maps were as effective as the network that was fed with raw data. This proves that the compression of the raw data into two maps was efficient, and that most of the information needed for our task was preserved.

Training a deep learning model requires many labeled examples, while in our study a relatively small MRI dataset was used. Increased performance was demonstrated using combinations of known principles such as the use of patch-based methods and standard geometric data augmentation that enriched the dataset. Using patches instead of entire images for training greatly increases the variability of the data. This compensates for the relatively small amount of data while still allowing the method to be generalized on external data. This was demonstrated in this study, where we showed the efficiency of the patch-based approach on data from CMT patients. The use of geometric data augmentations was crucial in this work. Standard augmentation techniques, on the other hand, such as color and brightness modifications were not considered, since muscle dystrophies are mainly identified based on image contrast, while contrast augmentations might confuse the networks leading to false predictions.

The second stage was intended for splitting the muscle region into viable muscle and infiltrated fat. The lack of accurate segmentation urged us to look for a weakly supervised method. The selection of a patch-based approach was essential considering the small amount of available data. The selection of architecture and loss function, which incorporated a triplet loss constraint, demonstrated its superiority compared with other clustering methods that were investigated in this work, especially DCAEDC which uses clustering loss. The results showed that the triplet loss boosts the results of classification and surpasses the deep convolutional auto-encoder with the embedding clustering that is widely used. To wrap the two stages together, an application of disease quantification and clustering to three disease states was demonstrated. Linear regression analysis demonstrated the agreement between our quantification score and the GT. The distinct margins between the three stages provide evidence that the selected methods can detect the disease state with small errors.

The robustness of the proposed approach was particularly apparent in cases where the MR images reflected high disease heterogeneity as well as when facing MRI artifacts that bias the raw data. As was demonstrated in Figure 7, an almost identical performance was achieved when training the network with and without bias field correction. This stability can be attributed to the use of different types of inputs for training the networks including combinations of quantitative T_2_ maps, PD maps, and raw images as well as a wide range of disease severity levels—all of which improved the accuracy and consistency of the results.

The method was tested on patients with mild, moderate, and severe muscular dystrophies demonstrating the method’s ability to identify the borders between tissues, even when the disease was in an advanced stage. The fraction of the infiltrated fat was also calculated, and a high correlation between this index and the disease severity was shown. The method was evaluated on another type of muscle dystrophy, the Charcot–Marie–Tooth (CMT) disease, suggesting that this approach is applicable to a range of muscle dystrophies exhibiting similar pattern of fatty infiltration.

To assess the disease’s severity, radiologists typically examine T_2_-weighted data: an image is collected at one predefined echo time, when the contrast is optimized to best highlight the disease. The disadvantages of this qualitative approach are that the estimation is reliant only on one specific echo-time. In the current work, on the contrary, the disease was estimated by looking at quantitative maps generated from full series of echo times. Thus, it is possible to synthesize any contrast level offline, greatly facilitating accurate prediction of the disease’s state. Furthermore, the EMC algorithm used to generate the T_2_ and PD maps relies on precise Bloch simulations of the experimental protocol timing diagram, radiofrequency (RF) pulse shapes, and gradient pulses, thereby producing accurate values that are stable across vendors and scan setting [32,40,41].

Many previous works quantified a disease state based on high-resolution T_1_, Dixon, and other MRI modalities. Methods include active contours, fuzzy c-means, k-means, and other conventional techniques. These methods are promising for the mild and moderate disease stages given high-resolution MR images, where the fine lines can be seen using the naked eye. However, they are challenged in severe disease levels, while deep-learning-based methods provide improved performance as shown in this study.

In this work, fat infiltration was estimated by considering multiple leg muscles as a single region. Future studies can include segmenting specific muscles and quantifying the fat infiltration in each muscle separately. To address the small data limitation, future work can also use the generative models in order to generate more diseased MRI images and augment the number of images. Lastly, future research can also include tracking the disease state over time using the quantitative biomarker calculated in this work. Fat infiltration into muscles and progressive loss of muscle tissue are associated with several pathologies of the peripheral nervous system that can benefit from the quantitative biomarker presented in this study.

## Figures and Tables

**Table 1:** Evaluation of the FCN muscle segmentation dice metric across different types of input data for three disease severity levels: mild, moderate, and severe. An overall Dice score was also calculated by combining all severity levels (combined) and by using a leave-one-patient-out (LOPO) procedure (pp: preprocessing; w/: with; w/o: without).

Input	Dice Coefficient [%]							
	T_2_ + PD		T_2_		PD		Raw Data	Aseg [36]
	w/ pp	w/o pp	w/ pp	w/o pp	w/ pp	w/o pp		
Mild	97.1	97.3	96.2	95.8	97.4	97.0	96.0	75.3
Moderate	92.6	95.9	88.3	95.9	96.2	96.0	97.5	65.0
Severe	96.4	94.9	94.0	93.8	96.2	96.0	95.8	57.5
Combined	95.6	95.8	93.1	94.8	96.4	96.2	96.3	64.4
LOPO	95.9	95.7	95.0	95.3	95.2	94.3	--	--

**Table 2:** Evaluation of the suggested FCN classification into viable muscle and IMAT in comparison to other common clustering approaches. As can be seen, our DCAETL + k-means approach produced the most accurate segmentation results—both in identifying viable muscle and IMAT. ACC was calculated based on Equation (10). See Section 2.6.3 for more detailed information on each evaluation metric.

Method	Viable Muscle Dice [%]	IMATDice [%]	ACC [%]	NMI [%]	ARI [%]
**k-means**	87.7	90.6	90.6	56.5	65.4
**DCAE + k-means**	89.5	91.5	91.7	58.6	69.2
**DCAEDC**	90.5	92.3	92.6	61.6	72.1
**DCAETL + k-means**	91.1	93.3	93.3	62.7	74.6

**Table 3:** Evaluation of ground-truth vs. FCN-based quantification fat infiltration for patients suffering from mild, moderate, and severe dysferlinopathy and CMT diseases.

Pathology	Disease Severity	GT [%]	Prediction [%]
**Dyspherlinopathy**	Mild	16.5 ± 6.4	19.5 ± 5.2
	Moderate	43.7 ± 8.1	36.7 ± 7.5
	Severe	87.1 ± 9.8	74.1 ± 7.6
**CMT**	Mild	15.2 ± 6.8	17.5 ± 5.7
	Moderate	45.5 ± 9.4	39.9 ± 8.6
	Severe	--	--

## Data Availability

Not applicable.

## Supplementary Materials

The following supporting information can be downloaded at: https://www.mdpi.com/article/10.3390/bioengineering9070315/s1, Figure S1: The architecture of U-net used for muscle-region segmentation; Figure S2: Data used for training the deep neural network.
