# Supplementary material for: Quantification of Intra-Muscular Adipose Infiltration in Calf/Thigh MRI Using Fully and Weakly Supervised Semantic Segmentation
Source: Bioengineering (Basel). 2022 Jul 14;9(7):315. doi: 10.3390/bioengineering9070315 (PMC9312115; doi:10.3390/bioengineering9070315)
Supplement: Supplementary file 1 [file bioengineering-09-00315-s001.zip › bioengineering-1764584 sp.pdf]

bioengineering

Supplementary Materials

Quantification of Intra-Muscular Adipose Infiltration in Calf/Thigh MRI Using Fully and Weakly Supervised Semantic Segmentation

Rula Amer [1], Jannette Nassar [1], Amira Trabelsi [2], David Bendahan [2], Hayit Greenspan [1,*] and Noam Ben-Eliezer [1,3,4,*]

[1] Department of Biomedical Engineering, Tel Aviv University, Tel Aviv 6139001, Israel; rolaamer@mail.tau.ac.il (R.A.); jannette2210@gmail.com (J.N.)
[2] Center for Magnetic Resonance in Biology and Medicine, Aix Marseille University, 13007 Marseille, France; amira.trabelsi.tn@gmail.com (A.T.); david.bendahan@univ-amu.fr (D.B.)
[3] Sagol School of Neuroscience, Tel-Aviv University, Tel Aviv, Israel
[4] Center for Advanced Imaging Innovation and Research (CAI2R), New York University School of Medicine, New York, NY 10016, USA
* Correspondence: noambe@tauex.tau.ac.il (N.B.E.); Tel.: +972-3-6409388

Citation: Amer, R.; Nassar, J.; Trabelsi, A.; Bendahan, D.; Greenspan, H.; Ben-Eliezer, N. Quantification of Intra-Muscular Adipose Infiltration in Calf/Thigh MRI Using Fully and Weakly Supervised Semantic Segmentation. *Bioengineering* **2022**, *9*, x. https://doi.org/10.3390/xxxxx

Academic Editor: Zhen Cheng

Received: 25 May 2022
Accepted: 8 July 2022
Published: date

Publisher's Note: MDPI stays neutral with regard to jurisdictional claims in published maps and institutional affiliations.

Copyright: © 2022 by the authors. Submitted for possible open access publication under the terms and conditions of the Creative Commons Attribution (CC BY) license (https://creativecommons.org/licenses/by/4.0/).

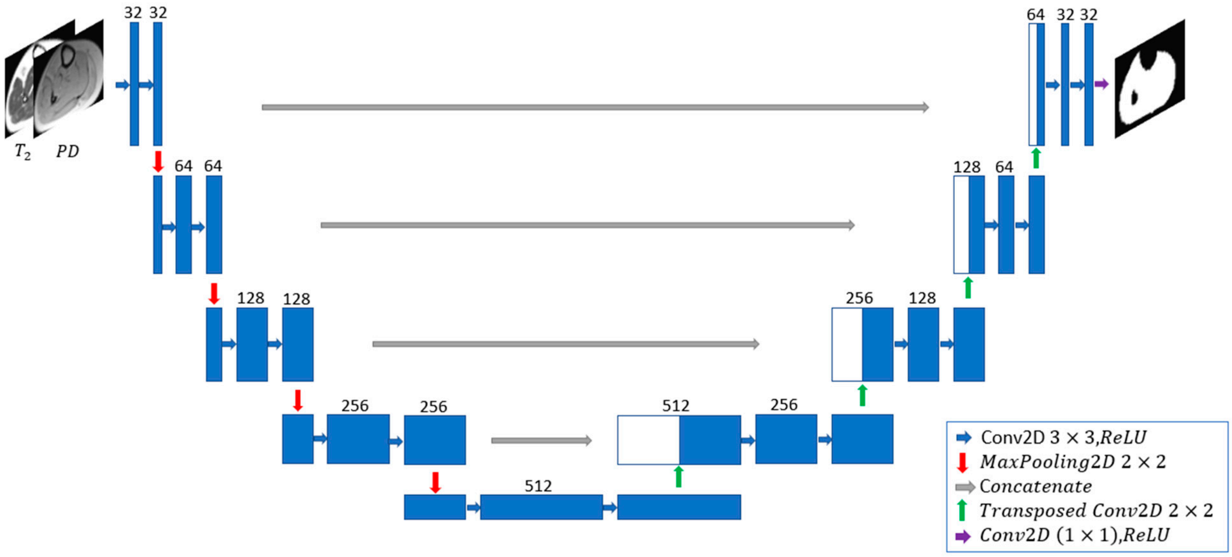

Figure S1. The architecture of U-net used for muscle-region segmentation.

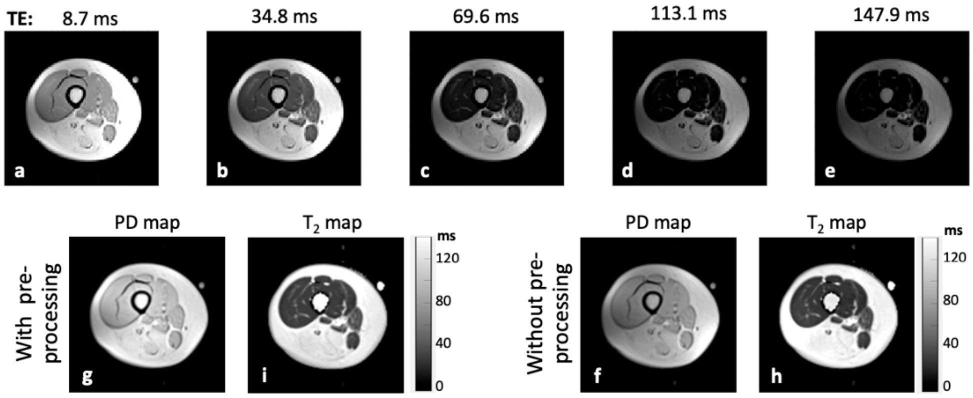

Figure S2. Data used for training the deep neural network. **(a-e)** Five representative raw T_2-weighted images from 1st, 4th, 8th, 13th, and 17th echo times (TEs). **(f-g)** Quantitative proton density maps [A.U.]. **(h-i)** Quantitative T_2 maps [ms]. Seven different combinations of thie data were used as inputs to train the network. (i) T_2 & PD maps w/o pre-processing; (ii) T_2 & PD maps w/ pre-processing; (iii / iv) Unprocessed / processed T_2 maps; (v / vi) Unprocessed / processed PD maps; (vii) Full set of raw T_2-weighted images (from all TEs).
